# Conformity to Bergmann's rule in birds depends on nest design and migration

**DOI:** 10.1002/ece3.8034

**Published:** 2021-08-23

**Authors:** Mark C. Mainwaring, Sally E. Street

**Affiliations:** ^1^ Field Research Station at Fort Missoula Division of Biological Sciences University of Montana Missoula MT USA; ^2^ Department of Anthropology Durham University Durham UK

**Keywords:** Bergmann's rule, migration, nest building, niche construction, phylogenetic comparative methods

## Abstract

Ecogeographical rules attempt to explain large‐scale spatial patterns in biological traits. One of the most enduring examples is Bergmann's rule, which states that species should be larger in colder climates due to the thermoregulatory advantages of larger body size. Support for Bergmann's rule, however, is not consistent across taxonomic groups, raising questions about what factors may moderate its effect. Behavior may play a crucial, yet so far underexplored, role in mediating the extent to which species are subject to environmental selection pressures in colder climates. Here, we tested the hypothesis that nest design and migration influence conformity to Bergmann's rule in a phylogenetic comparative analysis of the birds of the Western Palearctic, a group encompassing dramatic variation in both climate and body mass. We predicted that migratory species and those with more protected nest designs would conform less to the rule than sedentary species and those with more exposed nests. We find that sedentary, but not short‐ or long‐distance migrating, species are larger in colder climates. Among sedentary species, conformity to Bergmann's rule depends, further, on nest design: Species with open nests, in which parents and offspring are most exposed to adverse climatic conditions during breeding, conform most strongly to the rule. Our findings suggest that enclosed nests and migration enable small birds to breed in colder environments than their body size would otherwise allow. Therefore, we conclude that behavior can substantially modify species’ responses to environmental selection pressures.

## Graphical abstract

Bergmann's rule has received mixed support from empirical studies and here, we examined if behavior may moderate its effect. We show that amongst European birds, conformity to Bergmann's rule depends on migratory behavior and the design of nests.

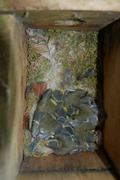

## 1 INTRODUCTION

Ecogeographical rules seek to characterize and explain spatial patterns in biological traits (Gaston et al., 2008). Bergmann's rule, which posits that species’ body mass increases in colder climates (Bergmann, 1847), is a classic example, yet the general validity of this principle and the biological mechanisms underpinning it remain controversial (Blackburn et al., 1999; Gaston et al., 2008). Bergmann originally proposed that among homeothermic species, body mass should be larger at more northerly latitudes because larger‐bodied organisms have a lower surface area‐to‐volume ratio and thus a greater ability to conserve heat (Bergmann, 1847; Blackburn et al., 1999). Several alternative mechanisms have since been proposed, however, such as a greater resilience to starvation in larger‐bodied species living in harsher climates (Blackburn et al., 1999; Blackburn & Hawkins, 2004). Regardless of the precise mechanism(s) underpinning Bergmann's rule, multiple global‐scale analyses show empirical support for its fundamental prediction that body mass increases in colder temperatures, including across bird species (Olson et al., 2009), mammal species (Clauss et al., 2013), and even human populations (Foster & Collard, 2013).

However, Bergmann's rule is far from universally supported. Despite an overall negative correlation between body mass and environmental temperature across bird and mammal species, only a minority of individual avian and mammalian orders conform to the rule (Clauss et al., 2013; Olson et al., 2009). Exceptions to Bergmann's rule include rodents, a highly speciose order accounting for ~40% of mammalian species (Alhajeri & Steppan, 2016). Evidently, many small‐bodied species are able to survive and reproduce in very cold climates. For example, the snow bunting (*Plectrophenax nivalis*) is able to breed in the Scottish highlands despite weighing only 28–50 g (RSPB, 2020) while the similarly sized white‐winged diuca finch (*Idiopsar speculifer*) breeds at altitudes of >5,000 m in the high Andes and, remarkably, builds its nest directly onto the ice (Schulenberg, 2020). Such compelling exceptions raise questions about what influences conformity to Bergmann's rule across diverse taxa and how some small‐bodied species meet the thermoregulatory challenges of surviving in extremely cold climates.

Behavior may be an important, yet relatively underinvestigated, mediator of conformity to Bergmann's rule. In particular, behaviors that limit the extent to which species are exposed to adverse climatic conditions may buffer them against selection for larger body sizes in colder climates, an effect termed “counteractive niche construction” by proponents of niche construction theory (Odling‐Smee et al., 2003, p. 288–9). If so, we should expect taxonomic groups characterized by such behaviors to correspond less to Bergmann's rule than others (Odling‐Smee et al., 2003, p. 288–9). The construction of nests, burrows, and other shelters by animals is likely one such behavior, as these structures can create stable microclimates that protect their inhabitants from extreme environmental conditions, thereby extending an organisms’ “control” over the environmental conditions to which it is exposed (Hansell, 2005).

Birds’ nests, although usually temporary structures built solely for breeding purposes, may offer protection from the adverse effects of colder temperatures for developing young and brooding parents (Mainwaring et al., 2014; Martin et al., 2017). Studies of single species suggest that both building enclosed nest structures (Lamprecht & Schmolz, 2004) and selecting naturally enclosed nest locations (Rhodes et al., 2009) reduce heat loss and stabilize temperatures inside the nest, likely with important benefits for offspring development and parental energy expenditure (Mainwaring et al., 2014; Martin et al., 2017). Protected nest designs, therefore, may allow small birds to breed in colder climates than they would otherwise be able to given their size. The idea that nest design might mediate conformity to Bergmann's rule has so far, however, received limited attention. When applied to variation in body mass within species (James, 1970), species with open nest designs are no more likely to conform to Bergmann's rule than those with enclosed nest designs (Meiri & Dayan, 2003). However, this prediction has not yet been tested at the interspecific level in birds, where variation in body mass far exceeds that at the intraspecific level (Blackburn et al., 1999).

Along with nest design, migration also likely has a strong effect on the extent to which birds conform to Bergmann's rule. Migrating birds can escape entirely from exposure to the coldest winter temperatures in temperate climates and therefore may be less subject to selection for larger body size compared with resident species that remain at higher latitudes year‐round (Ashton, 2002; Meiri & Dayan, 2003). Prior studies of Bergmann's rule at the intraspecific level (James, 1970) find some support for the prediction that sedentary species are more likely than migratory species to conform to the rule, but this effect is not entirely consistent across different samples and methodologies (Ashton, 2002; Meiri & Dayan, 2003). Like nest design, the potential effect of migration on conformity to Bergmann's rule at the interspecific level has yet to be investigated, as do potential interactions between the two behaviors.

Here, we investigate the potential role of nest design and migration as factors influencing conformity to Bergmann's rule in a phylogenetic comparative analysis of Western Palearctic birds. We focus on this region due to the dramatic variation in both body size and environmental conditions over large‐scale latitudinal gradients in the Northern Hemisphere, in which ambient temperatures are considerably colder in northerly than southerly regions. We hypothesize that sedentary species and those with more exposed nest designs conform to Bergmann's rule more strongly than migratory species and those with more protected nest designs. We thus predict that large body size should be more strongly associated with higher latitudes and colder temperatures for species with open compared with enclosed nest designs and for sedentary more than migratory species. Since nest structure and location may both influence exposure to climatic conditions (Lamprecht & Schmolz, 2004; Rhodes et al., 2009), we incorporate features of both nest structure and location when classifying species’ nest design, as well as examining their individual effects. If both nest design and migration have important effects on conformity to Bergmann's rule, we ought to find support for the predicted effects of nest design within both sedentary and migratory species and for the effects of migration within both open and enclosed nesting species. Alternatively, if either nest design or migration is the more important mediator of conformity to Bergmann's rule, one should override the effect of the other, and thus, we should find either no effect of nest design among migratory species or no effect of migration among enclosed nesting species. To test predictions, we fit phylogenetic regression models in which the slope of body mass on latitude or temperature was allowed to differ between species with varying nest designs and/or migratory strategies.

## 2 MATERIALS AND METHODS

### 2.1 Data collection

To test predictions, we compiled data on nest design, migration, body mass, breeding range temperature, and breeding latitude from online and literature databases (see below for details). After matching species from different datasets and with the phylogeny (Jetz et al., 2012), our sample sizes for analyses were *n* = 515 and *n* = 513 for those based on temperature and latitude, respectively.

### 2.2 Body size

We quantified species’ body sizes as the mean body mass (in grams) of males and females during the breeding season, preferring estimates from the UK (where appropriate) due to larger sample sizes, using data from the Birds of the Western Palearctic (Cramp, 1985, 1988, 1992; Cramp & Perrins, 1993, 1994a, 1994b; Cramp & Simmons, 1977, 1980, 1983). Body masses of unknown sex were used where body mass was not reported separately for males and females, following, for example, Møller et al. (2010).

### 2.3 Nest design

We classified species’ nest designs based on descriptions in the Birds of the Western Palearctic book series (Cramp, 1985, 1988, 1992; Cramp & Perrins, 1993, 1994a, 1994b; Cramp & Simmons, 1977, 1980, 1983). When categorizing nests for our study, it was important to consider not only the design of the structure built by the bird itself but additionally the nest site, because the location in which the nest is built also strongly affects its exposure to climatic conditions (Mainwaring et al., 2014). For example, open cup‐shaped nests built in vegetation should be more exposed to environmental conditions than open cup‐shaped nests built inside tree cavities (von Haartman, 1957). Therefore, we considered both aspects of the structure and location of birds’ nests to produce a single, biologically relevant “nest design” factor as follows. Nest structure was classified following Hansell (2000, figure 3.2) as either cup, plate, scrape, bed, dome, dome and tube, or burrow, while nest location was classified as open, semi‐open, or enclosed in which open refers to fully exposed nest sites (such as waders nesting on bare ground), semi‐open refers to those nests that are largely concealed from all sides by, for example, being located in dense vegetation, and enclosed refers to nests in tree cavities and alike (Alerstam & Hogstedt, 1981; von Haartman, 1957; Hansell, 2000). We then combined aspects of both structure and location to classify species’ overall nest design as either open, semi‐open, or enclosed (Table S1). We considered as “open” nest designs only open nest structures (cup, plate, scrape, or bed nests) built in open locations. We considered as “enclosed” nests both nests of any structure located inside cavities, and enclosed nest structures (dome, dome and tube, or burrow) built in any location. Finally, we treated open nest structures (cup, plate, scrape, or bed nests) built in “semi‐open” locations as “semi‐open” nests, an intermediate state between fully open and fully enclosed nest designs. In order to tease apart the effects of nest structure and location, however, we also ran analyses treating nest structure and location as separate variables (presented in Supporting Information).

### 2.4 Migration

Using data from the Birds of the Western Palearctic (Cramp, 1985, 1988, 1992; Cramp & Perrins, 1993, 1994a, 1994b; Cramp & Simmons, 1977, 1980, 1983), we categorized species’ migratory behavior, distinguishing between sedentary (nonmigratory) species, short‐distance migrants, and long‐distance migrants. Sedentary species were classified as those that remain in the same area year‐round and are thus residents, while short‐distance migrants migrate south each autumn to overwinter either in southern Europe or in northern Africa, and long‐distance migrants migrate south each autumn to overwinter in sub‐Saharan Africa.

### 2.5 Breeding climate

Bergmann's rule has generally been tested using measures of climatic conditions across a species’ entire range. However, since our predictions concern how body mass may be affected by exposure to climatic conditions while breeding, we investigated relationships between species’ body mass and climatic conditions of the breeding range specifically. We used two variables to capture climatic conditions in the breeding range: breeding range latitude and breeding range temperature. We obtained northernmost and southernmost latitudes of the breeding ranges for each species from distribution maps in the Birds of the Western Palearctic book series (Cramp, 1985, 1988, 1992; Cramp & Perrins, 1993, 1994a, 1994b; Cramp & Simmons, 1977, 1980, 1983). For analyses, we used a single latitudinal measure, “breeding latitude midpoint,” taken as the mean of the northernmost and southernmost breeding latitudes. To estimate breeding range temperature, we matched estimates of the mean temperature of the warmest quarter (“BIO10”) from WorldClim 2 (Fick & Hijmans, 2017) with species’ ranges from BirdLife International (BirdLife International & Handbook of the Birds of the World, 2018) using functions from the R packages “rgdal” (Bivand et al., 2018) and “letsR” (Vilela & Villalobos, 2015). Species’ ranges were converted to presence–absence matrices with 0.5‐degree grid cell resolution (~55 km at the equator), counting a species as present if its range covered 10% or more of a cell. We excluded uncertain records (presence codes 2 = “probably extant,” 3 = “possibly extant,” and 6 = “presence uncertain”) and records from outside of species’ native ranges (all except origin code 1 = “native” and 2 = “reintroduced”). To limit records to ranges in which birds may breed, we selected only records from the birds’ resident or breeding season ranges (season codes 1 = “resident” or 2 = “breeding season”), thereby excluding nonbreeding season and passage ranges, and records of uncertain seasonality (season codes 3 = “nonbreeding season,” 4 = “passage,” and 5 = “seasonal occurrence uncertain”). We obtained temperature data at 10 min of a degree resolution, matching it to each grid cell where a species was present. Since the climatic data were higher resolution than the presence–absence matrix, we averaged climatic data across cells at the coarser 0.5‐degree resolution to match the presence–absence matrix. Finally, we summarized breeding range temperature at the species level by taking the mean annual temperature across all occupied cells in each species’ presence–absence matrix.

### 2.6 Statistical analyses

We tested predictions using Bayesian phylogenetic generalized linear mixed models and phylogenetic generalized least squares regression, implemented in the MCMCglmm R package (Hadfield, 2010) and BayesTraits software (Meade & Pagel, 2016; Pagel, 1999; Pagel et al., 2004), respectively. To test for Bergmann's rule across the whole sample of species, we fit body mass as the outcome variable, predicted by either breeding range latitude or temperature. To investigate whether conformity to Bergmann's rule is affected by nesting variables and migration, we included an interaction term allowing slopes of body mass on latitude or temperature to vary between species with different nest characteristics and/or migratory strategies. For models incorporating interactions, sample sizes were sufficient such that there were at least 10 species included for every slope estimated. Body mass was log10‐transformed to correct for a strong positive skew, while breeding latitude and temperature were roughly normally distributed and so were left untransformed.

Accounting for phylogenetic nonindependence is essential in cross‐species comparative analyses to avoid pseudoreplication and biased parameter estimates (Freckleton et al., 2002). We obtained trees from a comprehensive global bird phylogeny (Jetz et al., 2012), and selected a version constructed using only species with molecular data, based on the Hackett et al. (2008) “backbone” phylogeny. For the majority of our analyses, we used a single maximum clade credibility (MCC) phylogeny based on a posterior sample of 10,000 trees, created with TreeAnnotator (Drummond et al., 2012). However, to ensure analyses were robust to phylogenetic uncertainty, we repeated one of our main analyses across a posterior distribution of 3,000 trees in BayesTraits (Meade & Pagel, 2016; Pagel, 1999). This approach uses MCMC to estimate model parameters across the posterior tree distribution, thereby incorporating both model and phylogenetic uncertainty into the results (Pagel et al., 2004). We found qualitatively identical results, both when sampling trees in proportion to their likelihood and when visiting each tree for an equal number of iterations (Table S2). Therefore, we are confident that our results are not substantially affected by phylogenetic uncertainty. For MCMCglmm analyses, we quantified the influence of phylogeny on our results by estimating heritability (*h* ^2^), the proportion of residual variance attributable to phylogenetic relationships, equivalent to Pagel's *λ* for PGLS regression (Hadfield & Nakagawa, 2010). Like Pagel's λ, *h* ^2^ varies from 0, equivalent to an ordinary nonphylogenetic regression with a random error structure, to 1, where the covariation in residual errors is directly proportional to phylogenetic relationships, assuming a Brownian motion model of trait evolution (Freckleton et al., 2002; Pagel, 1999).

For all models, we ran MCMC chains of sufficient length to obtain effective sample sizes of at least 500 for all model parameters (MCMCglmm = 201,000 iterations, sampling every 100 iterations, with a burn‐in period of 1,000 iterations; BayesTraits = 5,050,000 iterations, sampling every 1,000 iterations, with a burn‐in of 50,000 iterations). For MCMCglmm analyses, we used default, diffuse normal priors for predictor variables (with mean = 0, variance = 10^8^) and commonly used inverse‐Wishart priors for the residual variance and phylogenetic random effect (with *V* = 1, *ν* = 0.002, equivalent to an inverse Gamma distribution with shape and scale parameters set to 0.001, which results in a near‐uniform distribution; Hadfield, 2019). For BayesTraits analyses, we used default minimally informative, uniform prior distributions for all parameters, with a range of −100 to 100 for fixed effects and 0 to 1 for Pagel's *λ* (Meade & Pagel, 2016). We chose to use minimally informative, near‐flat priors to avoid biasing our parameter estimates in any particular direction. Sensitivity analyses confirmed that our results are robust to alternative prior specifications (Figures S1 and S2). For every model, we ensured that chains had converged on the posterior distribution, that burn‐in periods were sufficient, and that chains did not have problematic levels of autocorrelation by confirming sufficient effective sample sizes and by visual examination of chain plots. For all parameter estimates, we reported means and 95% credible intervals from posterior distributions. Additionally, for each slope estimated, we reported a “pMCMC value” (the probability that the slope is zero), and for each model, we reported *R^2^* values as the proportion of total variance explained by the fixed effects (i.e., the predictor variables, “marginal” *R^2^*) and the proportion of total variance explained by both the fixed and random effects (i.e., the predictor variables and phylogeny, “conditional” *R* ^2^; Nakagawa & Schielzeth, 2013). We also report the proportion of remaining, nonphylogenetic variance explained by the fixed effects (calculated as marginal *R* ^2^/(1−(conditional *R* ^2^‐marginal *R* ^2^))). We calculated effect sizes for selected key variables by estimating predicted values of species’ body mass based on model coefficients. We exponentiated the predicted values to translate back from the log_10_ to the original data scale.

## 3 RESULTS

### 3.1 Support for Bergmann's rule

In support of Bergmann's rule, species’ body mass increases slightly with breeding range latitude (*β* = 0.002 [95% CI: >−0.001, 0.004], pMCMC = 0.116, *n* = 513, *h* ^2^ *= *0.989 [0.973, 0.998], marginal *R* ^2^ = 0.002, conditional *R* ^2^ = 0.989; Figure S3a) and decreases with breeding range temperature (*β* = −0.006 [−0.010, −0.001], pMCMC = 0.007, *n* = 515, *h* ^2^ = 0.989 [0.973, 0.998], marginal *R* ^2^ = 0.004, conditional *R* ^2^ = 0.989; Figure S3b) across the whole sample of birds of the Western Palearctic. *R^2^* values show that breeding range latitude and temperature account for less than 1% of the variation in body mass, while the vast majority (>98%) of variation in body mass is explained by the phylogenetic random effect (Table S3). Latitude and temperature, however, explain 14% and 27% of the remaining variance not explained by phylogeny, respectively (Table S3). Our models predict that average body mass increases from ~274 g to ~357 g across the full range of breeding latitudes represented in the data (16 to 80 degrees), and decreases from ~432 g to ~283 g from the lowest to the highest breeding range temperatures (<1°C to 32°C).

### 3.2 Effects of nest design and migration on conformity to Bergmann's rule

When incorporating interactions of nest design and breeding climate into models, we find that nest design affects conformity to Bergmann's rule (Table 1). Specifically, in semi‐open nesting species, we find similar effects to those found across the whole sample: Body mass increases slightly with breeding range latitude (*β* = 0.003 [−0.001, 0.006], pMCMC = 0.089; Table 1a) and decreases with breeding range temperature (*β* = −0.010 [−0.017, −0.003], pMCMC = 0.005; Table 1b). However, among open and enclosed nesting species, there is little to no relationship between body mass and either latitude or temperature (pMCMCs ≥ 0.2; Table 1). Repeating analyses for nest location and structure separately suggests that this pattern is driven primarily by nest location rather than structure (Tables S4 and S5).

**TABLE 1:** Interaction of Bergmann's rule with nest design

Nest design	*β*	*β* 95% CI	pMCMC	*h* ^2^	*h* ^2^ 95% CI	Marg. *R* ^2^	Cond. *R* ^2^
(a) Latitude (*n* = 513 species)							
Open	>−0.001	[−0.003, 0.004]	0.988	0.987	[0.967, 0.998]	0.027	0.987
Semi‐open	0.003	[−0.001, 0.006]	0.089				
Enclosed	0.002	[−0.002, 0.007]	0.311				
(b) Temperature (*n* = 515 species)							
Open	−0.001	[−0.007, 0.006]	0.711	0.989	[0.972, 0.998]	0.031	0.990
Semi‐open	−0.010	[−0.017, −0.003]	0.005				
Enclosed	−0.006	[−0.016, 0.004]	0.238				

Note

*β*/*β* 95% CI/pMCMC = posterior mean regression slope estimates, 95% credible intervals, and pMCMC values for body mass on (a) latitude and (b) temperature, fitted for species with different nest designs. *h* ^2^/*h* ^2^ 95% CI = mean heritability (phylogenetic signal) and 95% credible intervals, marg.*R* ^2^ = marginal *R* ^2^, the proportion of variance in the dependent variable explained by the fixed effects, and cond. *R* ^2^ *= *conditional *R* ^2^, the proportion of variance in the dependent variable explained by both the fixed and random (phylogenetic) effects.

**FIGURE 1:**
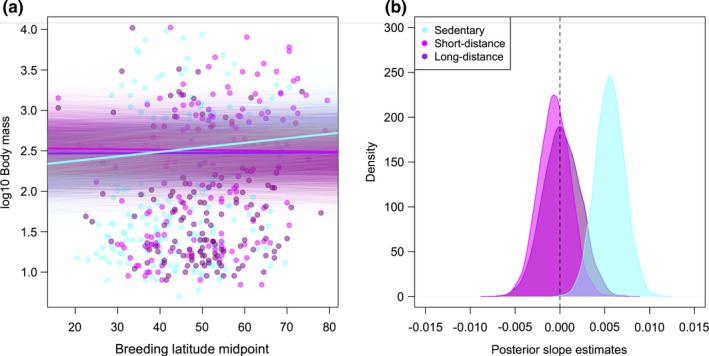
Interaction of Bergmann's rule with migration. (a) Species’ body mass against breeding latitude midpoint with different slopes fitted for sedentary, short‐distance migrating, and long‐distance migrating species. Mean slopes from posterior distributions are indicated by thick lines, while slopes from the entire posterior distributions are plotted as thinner, semi‐transparent lines. (b) Density plot showing posterior distributions of slope estimates for sedentary, short‐distance migrating, and long‐distance migrating species

When including interaction terms for migration and breeding climate, we find support for the predicted effect of migration on conformity to Bergmann's rule: Among sedentary species, body mass increases with breeding latitude (*β* = 0.006 [0.003, 0.009], pMCMC < 0.001; Table 2a, Figure 1) and decreases with breeding range temperature (*β* = −0.014 [−0.021, −0.008], pMCMC < 0.001; Table 2b), but this is not the case in short‐ or long‐distance migrants (pMCMCs ≥0.6; Table 2).

**TABLE 2:** Interaction of Bergmann's rule with migration

Migration	*β*	*β* 95% CI	pMCMC	*h* ^2^	*h* ^2^ 95% CI	Marg. *R* ^2^	Cond. *R* ^2^
(a) Latitude (*n* = 513 species)							
Sedentary	0.006	[0.003, 0.009]	<0.001	0.991	[0.976, 0.999]	0.007	0.991
Short distance	−0.001	[−0.004, 0.003]	0.711				
Long distance	<0.001	[−0.004, 0.004]	0.940				
(b) Temperature (*n* = 515 species)							
Sedentary	−0.014	[−0.021, −0.008]	<0.001	0.990	[0.974, 0.999]	0.010	0.991
Short distance	>−0.001	[−0.008, 0.005]	0.820				
Long distance	−0.002	[−0.012, 0.006]	0.626				

Note

*β*/*β* 95% CI/*β* pMCMC = posterior mean regression slope estimates, 95% credible intervals, and pMCMC values for body mass on (a) latitude and (b) temperature, fitted for species with different migratory behaviors. *h* ^2^/*h* ^2^ 95% CI = mean heritability (phylogenetic signal) and 95% credible intervals, marg.*R* ^2^ = marginal *R* ^2^, the proportion of variance in the dependent variable explained by the fixed effects, and cond. *R* ^2^ *= *conditional *R* ^2^, the proportion of variance in the dependent variable explained by both the fixed and random (phylogenetic) effects.

**FIGURE 2:**
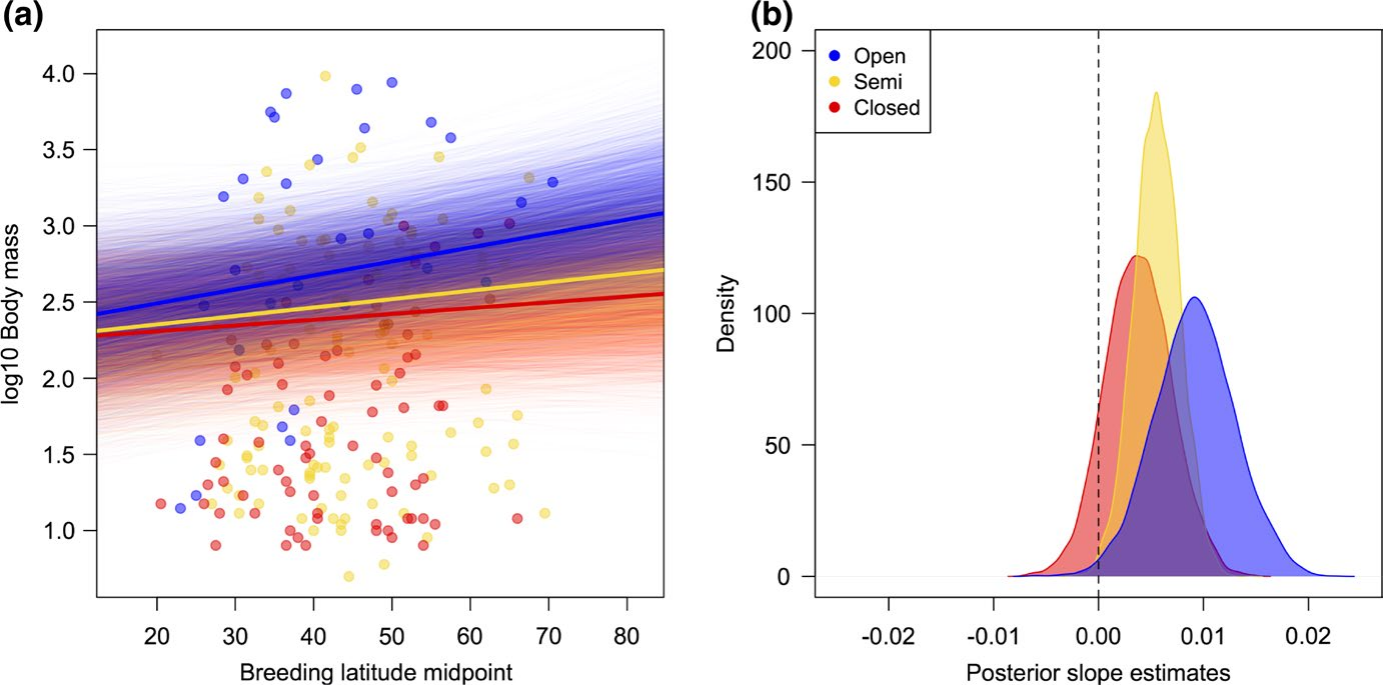
Interaction of Bergmann's rule with nest design within sedentary species. (a) Species’ body mass against breeding latitude midpoint with different slopes fitted for open, semi‐open, and enclosed nesting species, within sedentary species only. Mean slopes from posterior distributions are indicated by thick lines, while slopes from the entire posterior distributions are plotted as thinner, semi‐transparent lines. (b) Density plot showing posterior distributions of slope estimates for open, semi‐open, and enclosed nesting species, within sedentary species only

When including a three‐way interaction between breeding climate, nest design, and migration, we find the predicted effects of nest design on conformity to Bergmann's rule within sedentary species. Among sedentary species, body mass increases with breeding range latitude and decreases with breeding range temperature for open and semi‐open nesting species (pMCMCs ≤ 0.035), but far more weakly among enclosed nesting species (pMCMCs ≥ 0.1; Table 3, Figure 2). Short‐ and long‐distance migrating species do not conform to Bergmann's rule at all, regardless of nest design (pMCMCs ≥ 0.4; Table 3). *R* ^2^ values show that climatic, nesting, and migratory interactions together explain ~3% of the total variance in body mass, but >70% of the remaining variance in body mass not explained by phylogeny (Table S3). When rerunning analyses separating the effects of nest structure and location, we find similar patterns for both nest structure (Table S6) and location (Table S7). Using predicted values to illustrate effect sizes, we find that for sedentary species with open nests, the model predicts, on average, an increase of ~12 g in body mass (from ~565 to ~577 g) for an increase of one degree of latitude, assuming a species breeding at the median latitude (48 degrees). In contrast, for sedentary species with enclosed nests breeding at the same latitude, an increase of one degree is predicted to increase body mass only by ~3 g (from ~266 g to 269 g), while for a long‐distance migrating species with an open nest, no increase is predicted at all (remaining at ~377 g).

**TABLE 3:** Interaction of Bergmann's rule with migration and nest design

Migration	Nest design	*β*	*β* 95% CI	pMCMC	*h* ^2^	*h* ^2^ 95% CI	Marg. *R* ^2^	Cond. *R* ^2^
(a) Latitude (*n* = 513 species)								
Sedentary	Open	0.009	[0.001, 0.016]	0.024	0.988	[0.971, 0.998]	0.031	0.989
	Semi	0.006	[0.002, 0.010]	0.007				
	Enclosed	0.004	[−0.002, 0.010]	0.253				
Short	Open	−0.002	[−0.006, 0.003]	0.537				
	Semi	0.001	[−0.004, 0.007]	0.720				
	Enclosed	−0.002	[−0.010, 0.006]	0.692				
Long	Open	<−0.001	[−0.007, 0.005]	0.907				
	Semi	−0.002	[−0.008, 0.004]	0.538				
	Enclosed	0.004	[−0.006, 0.014]	0.454				
(b) Temperature (*n* = 515 species)								
Sedentary	Open	−0.014	[−0.027, −0.001]	0.035	0.989	[0.970, 0.999]	0.035	0.989
	Semi	−0.016	[−0.025, −0.007]	<0.001				
	Enclosed	−0.010	[−0.022, 0.003]	0.136				
Short	Open	0.001	[−0.007, 0.010]	0.801				
	Semi	−0.004	[−0.015, 0.007]	0.473				
	Enclosed	<0.001	[−0.019, 0.018]	0.922				
Long	Open	−0.003	[−0.013, 0.009]	0.654				
	Semi	0.001	[−0.014, 0.016]	0.917				
	Enclosed	−0.004	[−0.030, 0.022]	0.770				

Note

*β*/*β* 95% CI/*β* pMCMC = posterior mean regression slope estimates, 95% credible intervals, and pMCMC values for body mass on (a) latitude and (b) temperature, fitted for species with different nest designs and migratory behaviors. *h* ^2^/*h* ^2^ 95% CI = mean heritability (phylogenetic signal) and 95% credible intervals, marg.*R* ^2^ = marginal *R* ^2^, the proportion of variance in the dependent variable explained by the fixed effects, and cond. *R* ^2^ = conditional *R* ^2^, the proportion of variance in the dependent variable explained by both the fixed and random (phylogenetic) effects.

## 4 DISCUSSION

We find that conformity to Bergmann's rule, which predicts that species inhabiting colder environments should have larger body sizes (Bergmann, 1847), is not uniform across birds of the Western Palearctic, but rather depends on migration and nest design. Specifically, body mass is unaffected by climatic conditions in the breeding range for both long‐ and short‐distance migrating species, while among nonmigrating species, conformity to Bergmann's rule depends on nest design. For sedentary species, we find the strongest relationships between body mass and climate across species with open nest designs, in which offspring and brooding parents are most exposed to adverse climatic conditions, followed by those with semi‐open nests, while body mass is most weakly associated with climate across species with enclosed nests. Our results suggest that smaller birds can adapt to colder climates not only via selection for larger body size, but also alternatively by migrating to avoid extreme winter temperatures, or by adopting more insulated, enclosed nest designs if they remain at higher latitudes all year‐round.

Our findings are consistent with a prediction by proponents of niche construction theory (NCT) that susceptibility to abiotic selection pressures can be moderated by species’ alteration of their environments through behavior, a process termed “counteractive niche construction” (Odling‐Smee et al., 2003, p. 288–9). In fact, in *Niche construction: the neglected process in evolution*, Odling‐Smee et al. (2003) specifically predicted that conformity to Bergmann's rule should be mediated by behavior that limits exposure to climatic conditions (Odling‐Smee et al., 2003, p. 288–9), as we find here. Our study, therefore, shows that NCT can make clear, testable predictions about the natural world, a point of contention between NCT advocates and skeptics (Scott‐Phillips et al., 2014). However, it is important to acknowledge that comparative analyses can only identify correlational rather than causal relationships (Nunn, 2011), and as such, we cannot rule out the possibility of alternative causal explanations for our findings. In this case, our results are equally compatible both with an explanation based in NCT, in which migration and nesting determine the environmental selection pressures to which a species is exposed, and with an explanation based in “standard” evolutionary theory, in which migration, enclosed nesting, and larger body sizes are alternative responses to environmental selection pressures. Therefore, while our results are consistent with a prediction of NCT, we note that we could have made the same predictions without it (Scott‐Phillips et al., 2014).

Multiple mechanisms have been proposed to underpin Bergmann's rule, including those unrelated to thermoregulation (Blackburn et al., 1999; Blackburn & Hawkins, 2004). For example, the “resource availability” hypothesis posits that a larger body mass has a selective advantage in colder, temperate climates due to increased fat storage and thus a greater resilience to seasonal fluctuations in food availability (Blackburn et al., 1999; Blackburn & Hawkins, 2004). Although comparative analyses cannot provide direct evidence of causal mechanisms (Nunn, 2011), our results strongly point toward heat conservation as the main explanation for latitudinal gradients in body mass in Western Palearctic birds. The greater conformity to Bergmann's rule among open‐nesting species that we identify is consistent with the idea that enclosed nests play an important role in maintaining temperatures favorable for offspring development and reducing the energetic costs of incubation (Mainwaring et al., 2014; Martin et al., 2017) in smaller‐bodied species breeding in colder climates. In contrast, the resource availability hypothesis would predict no such interaction between nest design and conformity to Bergmann's rule. Furthermore, across our models we find that breeding range temperature is a stronger predictor of body mass than breeding latitude, suggesting that the latitudinal gradients in body mass we identify are primarily reflective of variation in environmental temperature rather than resource availability or any other confounding factor.

A bird's nest “design” incorporates both structural features built by the bird itself and naturally occurring features of the chosen nest site (Mainwaring et al., 2014). In particular, a bird can create an enclosed nest either by building a roofed structure or by placing an open structure inside a naturally existing cavity, both of which may help maintain optimal temperatures inside the nest (Lamprecht & Schmolz, 2004; Rhodes et al., 2009). For our main analyses, we classified nest design based on both features of nest structure and location, while in supplementary analyses, we find that nest location is at least as important as structure in affecting conformity to Bergmann's rule. Therefore, nests placed in enclosed locations, such as inside tree cavities, appear just as effective in buffering small‐bodied species against exposure to colder conditions as those with roofs constructed from vegetation. These results demonstrate the importance of considering both features of nest structure and location when investigating the role nests may play in protecting their inhabitants from exposure to environmental hazards.

Along with nest design, we also find that migration is an important mediator of conformity to Bergmann's rule, consistent with some previous intraspecific analyses (Meiri & Dayan, 2003; but see Ashton, 2002). We find that body mass increases in colder temperatures only among sedentary species, while short‐ and long‐distance migrants do not conform to Bergmann's rule at all. These findings support the idea that migrating Western Palearctic species are completely buffered against selection for large body size in colder climates as they avoid exposure to the coldest winter temperatures at high latitudes by spending the nonbreeding season in warmer environments in southern Europe or Africa (Ashton, 2002; Meiri & Dayan, 2003). When incorporating both the effects of migration and nest design into our models, we find that migration has the stronger effect: There is no effect of nest design on conformity to Bergmann's rule among migratory species. Therefore, the thermoregulatory benefits of migration appear to override those of nest design, such that enclosed nests provide no additional thermoregulatory benefits for migratory, small‐bodied species. This is perhaps unsurprising because migration allows species to avoid extreme winter conditions altogether, while nest design can only affect exposure to environmental conditions for relatively limited periods of time during breeding.

In contrast to our findings, when applying Bergmann's rule to within‐species variation (James, 1970), a comparative study of 106 bird species found no effect of nest design on conformity to the rule: Body mass was no more likely to increase in colder climates within open‐nesting than enclosed nesting species (Meiri & Dayan, 2003). However, Bergmann's rule does not necessarily operate in the same way at intra‐ and interspecific scales, and latitudinal gradients in body size may be more likely to be detected in large‐scale interspecific studies such as ours, which capture far more variation in body mass and climatic conditions than intraspecific studies (Blackburn et al., 1999). Further, in contrast to this prior study, we investigated relationships between body mass and environmental conditions in birds’ breeding ranges specifically, rather than across their entire geographic ranges. Nest design is much more relevant to the former since birds’ nests are generally temporary structures built for the purposes of breeding only.

Our finding that enclosed nests buffer birds against exposure to extreme climatic conditions should generalize to other taxa in which species build similarly protective structures for breeding and shelter. To our knowledge, only one study has so far investigated this possibility in mammals, but found no support for the predicted effect of burrowing on conformity to Bergmann's rule (Alhajeri & Steppan, 2016). Rodent species living above ground do not exhibit stronger correlations between body mass and environmental temperature than underground‐dwelling species, and the study found little support for Bergmann's rule across rodents as a whole (Alhajeri & Steppan, 2016). It remains possible, however, that burrowing in mammals interacts with other traits, such as hibernation, in buffering species against adverse climates in a manner analogous to the combined role of migration and nesting we identify in birds. In our study, we did not find support for the predicted effect of nesting on conformity to Bergmann's rule across the whole sample, but rather only when accounting for the (stronger) effect of migration. Therefore, our findings highlight the potential for behavioral traits to act in combination with one another to influence species’ responses to environmental selection pressures in complex ways.

While our study illustrates the benefits of enclosed nests for protection from colder climatic conditions, two recent comparative analyses suggest that enclosed nests are primarily protective against exposure to hot and dry conditions. Among diverse geographic regions, the proportion of passerine species with enclosed nests is two to three times greater in tropical or Southern Hemisphere regions than in north temperate regions (Martin et al., 2017). Within Australia, meanwhile, the proportion of passerine species building domed nests increases in areas with hotter, drier climates, and less vegetative cover (Duursma et al., 2018). Direct comparisons between our results and these prior studies are challenging due to key methodological differences: These two studies are based on spatial rather than interspecific patterns and do not incorporate potential interactions with body mass, nor do they account for phylogenetic nonindependence. Discrepancies with our findings may also be partly explained by different approaches to classifying nest designs: In contrast to our study, these analyses did not count nests built in cavities as enclosed due to a focus solely on nest structure. In our sample, a substantial proportion (~20%) of species nest in cavities which are highly likely to provide effective protection against colder breeding environments in the Northern Hemisphere. Taken at face value, however, these differing results may suggest that protective effects of enclosed nests against extreme climatic conditions are variable across geographic regions. Enclosed nesting may only have the opportunity to evolve in response to colder climates within the Northern Hemisphere, which encompasses far more potential breeding range in temperate and polar climatic zones than the Southern Hemisphere.

While we identify clear effects of migration and nest design on conformity to Bergmann's rule in birds, our model *R^2^* values suggest that such behavioral traits explain relatively little of the overall variance in body mass (Table S3). This is to be expected, however, given that the vast majority (>98%) of variation in body mass in our sample is explained by phylogenetic history. Therefore, any additional effects of ecology or behavior on body mass must by necessity be small in terms of the total amount of variance explained (although potentially substantial in terms of the proportion of nonphylogenetic variance explained, as we find here). This does not mean that such effects are biologically insignificant, however, because even small changes in body mass may have substantial fitness consequences when amplified over multiple generations at evolutionary timescales (Møller & Jennions, 2002), particularly for relatively small species with energetically costly lifestyles (due to, e.g., endothermy and powered flight). Comparing marginal *R* ^2^ values for models with and without interaction terms suggests that interactions explain relatively little additional variance over and above main effects of nesting or migration on body mass (Table S3). However, the marked differences in slopes between species with differing nesting and migratory behaviors that we identify, in support of multiple specific a priori predictions (Tables 1, 2, 3, Figures 1 and 2), provide compelling evidence for the role of behavior in limiting species’ exposures to climatic extremes. The extent to which such results generalize to other traits and taxonomic groups would be a productive avenue for future research.

We have demonstrated that in Western Palearctic birds, body mass increases in colder climates as predicted by Bergmann's rule only in nonmigratory species breeding in exposed nests. Our findings are consistent with the idea that migration and enclosed nests compensate for greater thermoregulatory costs in smaller‐bodied birds, allowing then to breed in colder environments than expected for their body size. Further research could usefully examine how species’ modification of environments affects responses to environmental selection pressures across more diverse taxa, traits, and geographic regions, including across human populations. Other studies could also usefully examine how behavior may influence responses to latitudinal variation in climatic variables other than temperature, such as precipitation. Our work should also guide future experimental studies on the potentially mediating role of nesting behavior on the influence of climatic conditions on parental and offspring fitness to more effectively test causal hypotheses. We conclude that behavior, particularly migration, nest‐building, and nest‐site choice, mediates species’ responses to climatic selection pressures.

## CONFLICT OF INTEREST

None declared.

## AUTHOR CONTRIBUTIONS

**Mark C. Mainwaring:** Conceptualization (equal); Data curation (lead); Writing‐original draft (equal); Writing‐review & editing (equal). **Sally E. Street:** Conceptualization (equal); Formal analysis (lead); Writing‐original draft (equal); Writing‐review & editing (equal).

## Supporting information

Supplementary Material

## Data Availability

The data and code are available in Dryad: https://doi.org/10.5061/dryad.0vt4b8h0h.
