# Supplementary material for: Conformity to Bergmann's rule in birds depends on nest design and migration
Source: Ecol Evol. 2021 Aug 23;11(19):13118–27. doi: 10.1002/ece3.8034 (PMC8495809; doi:10.1002/ece3.8034)
Supplement: Supplementary file 1 — Supplementary Material [file ECE3-11-13118-s001.docx]

**SUPPORTING INFORMATION**

**Table S1. Nest design categorisation scheme.**

|  | **Open** | **Semi-open** | **Enclosed** |
| --- | --- | --- | --- |
| **Cup** | Open | Semi-open | Enclosed |
| **Plate** | Open | Semi-open | Enclosed |
| **Scrape** | Open | Semi-open | Enclosed |
| **Bed** | Open | Semi-open | Enclosed |
| **Dome** | Enclosed | Enclosed | Enclosed |
| **Dome & tube** | Enclosed | Enclosed | Enclosed |
| **Burrow** | Enclosed | Enclosed | Enclosed |

Scheme used to combine nest structure and location into a single nest design variable. Nest structure (rows) and nest location (columns) categories were combined to form a single nest design variable, capturing differing levels of exposure to environmental conditions influenced by both nest structure and location.

**Table S2. Incorporating phylogenetic uncertainty.**

a) Sampling trees in proportion to their likelihood (n=513 species)

| **Nest design** | **Mean *β*** | ***β* 95% CI** | **Mean *λ*** | ***λ* 95% CI** |
| --- | --- | --- | --- | --- |
| Open | <-0.001 | [-0.004, 0.003] | 0.970 | [0.952, 0.983] |
| Semi-open | 0.003 | [-0.001, 0.006] |  |  |
| Enclosed | 0.002 | [-0.002, 0.002] |  |  |

b) Visiting all trees for an equal number of iterations (n=513 species)

| **Nest design** | **Mean *β*** | ***β* 95% CI** | **Mean *λ*** | ***λ* 95% CI** |
| --- | --- | --- | --- | --- |
| Open | <0.001 | [-0.004, 0.004] | 0.958 | [0.936, 0.976] |
| Semi-open | 0.003 | [-0.001, 0.006] |  |  |
| Enclosed | 0.002 | [-0.003, 0.007] |  |  |

Results of one of the main analyses (body mass predicted by an interaction of breeding latitude and nest design) which was repeated incorporating phylogenetic uncertainty by running the model across a posterior distribution of 3000 trees in BayesTraits. Initially trees were sampled in proportion to their likelihood (a), but this resulted in poor mixing between trees, likely caused by wide variation in tree likelihood across the sample. Results are qualitatively identical when forcing the chain to visit all trees in the sample for an equal number (1000) of iterations (b). Mean *β* / *β* 95% CI = mean regression slope estimates and 95% credible intervals, mean *λ / λ* 95% CI = mean Pagel’s lambda (phylogenetic signal) estimates and 95% credible intervals, from posterior distributions.

**Table S3 Summary of R^2^ values for key models.**

a) Latitude (n=513 species)

| **Independent variables** | **Marginal R^2^** | **Conditional R^2^** | **Prop. fixed** |
| --- | --- | --- | --- |
| Latitude only | 0.002 [<0.001, 0.005] | 0.989 [0.976, 0.999] | 0.138 |
| Latitude + nest design | 0.025 [0.006, 0.045] | 0.986 [0.971, 0.999] | 0.650 |
| Latitude * nest design | 0.027 [0.007, 0.047] | 0.987 [0.973, 0.999] | 0.678 |
| Latitude + migration | 0.004 [<0.001, 0.008] | 0.988 [0.975, 0.999] | 0.234 |
| Latitude * migration | 0.007 [0.002, 0.012] | 0.991 [0.979, 0.999] | 0.424 |
| Latitude + nest design + migration | 0.027 [0.009, 0.042] | 0.985 [0.969, 0.999] | 0.643 |
| Latitude * nest design * migration | 0.031 [0.014, 0.051] | 0.989 [0.975, 0.999] | 0.729 |

a) Temperature (n=515 species)

| **Independent variables** | **Marginal R^2^** | **Conditional R^2^** | **Prop. fixed** |
| --- | --- | --- | --- |
| Temperature only | 0.004 [<0.001, 0.009] | 0.990 [0.977, 0.999] | 0.271 |
| Temperature + nest design | 0.029 [0.008, 0.050] | 0.986 [0.971, 0.999] | 0.674 |
| Temperature * nest design | 0.031 [0.011, 0.054] | 0.990 [0.976, 0.999] | 0.750 |
| Temperature + migration | 0.007 [<0.001, 0.013] | 0.988 [0.973, 0.999] | 0.349 |
| Temperature * migration | 0.010 [0.003, 0.018] | 0.991 [0.979, 0.999] | 0.521 |
| Temperature + nest design + migration | 0.030 [0.010, 0.051] | 0.985 [0.969, 0.999] | 0.665 |
| Temperature * nest design * migration | 0.035 [0.016, 0.057] | 0.989 [0.974, 0.999] | 0.764 |

Marginal and conditional R^2^ values for key models with 95% credible intervals, illustrating the proportion of variance in body mass by the fixed effects and fixed + random effects respectively. Prop. fixed = the proportion of non-phylogenetic variance explained by the fixed effects, calculated as marginal R^2^/(1-(conditional R^2^ – marginal R^2^)).

**Table S4. Bergmann’s rule and nest structure.**

1. Latitude (n=513 species)

| **Nest structure** | **Mean *β*** | ***β* 95% CI** | ***pMCMC*** | **Mean *h^2^*** | ***h^2^* 95% CI** |
| --- | --- | --- | --- | --- | --- |
| Open | 0.001 | [-0.001, 0.004] | 0.286 | 0.988 | [0.972, 0.998] |
| Enclosed | 0.005 | [-0.002, 0.011] | 0.167 |  |  |

1. Temperature (n=515 species)

| **Nest structure** | **Mean *β*** | ***β* 95% CI** | ***pMCMC*** | **Mean *h^2^*** | ***h^2^* 95% CI** |
| --- | --- | --- | --- | --- | --- |
| Open | -0.005 | [-0.009, <0.001] | 0.055 | 0.988 | [0.972, 0.998] |
| Enclosed | -0.014 | [-0.030, 0.003] | 0.091 |  |  |

Results of models allowing the slope of body mass on a) latitude or b) temperature to vary between species with different nest structures (open = cup, plate, scrape or bed, closed = dome, dome and tube or burrow), regardless of location. Mean *β* / *β* 95% CI / *pMCMC* = mean regression slope estimates, 95% credible intervals and pMCMC values, mean *h^2^ / h^2^* 95% CI = mean heritability (phylogenetic signal) and 95% credible intervals.

**Table S5. Bergmann’s rule and nest location.**

1. Latitude (n=513 species)

| **Nest location** | **Mean *β*** | ***β* 95% CI** | ***pMCMC*** | **Mean *h^2^*** | ***h^2^* 95% CI** |
| --- | --- | --- | --- | --- | --- |
| Open | <-0.001 | [-0.004, 0.004] | 0.990 | 0.987 | [0.969, 0.998] |
| Semi-open | 0.003 | [<-0.001, 0.006] | 0.065 |  |  |
| Enclosed | 0.002 | [-0.004, 0.008] | 0.458 |  |  |

1. Temperature (n=515 species)

| **Nest location** | **Mean *β*** | ***β* 95% CI** | ***pMCMC*** | **Mean *h^2^*** | ***h^2^* 95% CI** |
| --- | --- | --- | --- | --- | --- |
| Open | -0.001 | [-0.007, 0.006] | 0.721 | 0.989 | [0.972, 0.999] |
| Semi-open | -0.010 | [-0.016,-0.004] | 0.001 |  |  |
| Enclosed | -0.005 | [-0.017, 0.007] | 0.444 |  |  |

Results of models allowing the slope of body mass on a) latitude or b) temperature to vary between species with different nest locations (open, semi-open or enclosed), regardless of structure. Mean *β* / *β* 95% CI / *pMCMC* = mean regression slope estimates, 95% credible intervals and pMCMC values, mean *h^2^ / h^2^* 95% CI = mean heritability (phylogenetic signal) and 95% credible intervals.

**Table S6. Interaction of Bergmann’s rule with migration and nest structure.**

1. Latitude (n=513 species)

| **Migration** | **Nest design** | **Mean *β*** | ***β* 95% CI** | ***pMCMC*** | **Mean *h^2^*** | ***h^2^* 95% CI** |
| --- | --- | --- | --- | --- | --- | --- |
| Sedentary | Open | 0.005 | [0.002, 0.008] | 0.006 | 0.990 | [0.972, 0.998] |
|  | Enclosed | 0.006 | [-0.005, 0.017] | 0.312 |  |  |
| Short | Open | <-0.001 | [-0.004, 0.003] | 0.727 |  |  |
|  | Enclosed | -0.004 | [-0.019, 0.010] | 0.610 |  |  |
| Long | Open | -0.001 | [-0.005, 0.004] | 0.704 |  |  |
|  | Enclosed | 0.007 | [-0.005, 0.018] | 0.243 |  |  |

1. Temperature (n=515 species)

| **Migration** | **Nest design** | **Mean *β*** | ***β* 95% CI** | ***pMCMC*** | **Mean *h^2^*** | ***h^2^* 95% CI** |
| --- | --- | --- | --- | --- | --- | --- |
| Sedentary | Open | -0.013 | [-0.019, -0.006] | <0.001 | 0.989 | [0.973, 0.999] |
|  | Enclosed | -0.021 | [-0.044, 0.005] | 0.113 |  |  |
| Short | Open | <-0.001 | [-0.007, 0.006] | 0.825 |  |  |
|  | Enclosed | 0.003 | [-0.029, 0.037] | 0.860 |  |  |
| Long | Open | -0.001 | [-0.011, 0.008] | 0.8774 |  |  |
|  | Enclosed | -0.012 | [-0.048, 0.018] | 0.490 |  |  |

Results of models allowing the slope of body mass on a) latitude or b) temperature to vary between species with different migratory strategies (sedentary, short- or long-distance) and nest structures (open = cup, plate, scrape or bed, closed = dome, dome and tube or burrow). Mean *β* / *β* 95% CI / *pMCMC* = mean regression slope estimates, 95% credible intervals and pMCMC values, mean *h^2^ / h^2^* 95% CI = mean heritability (phylogenetic signal) and 95% credible intervals.

**Table S7. Interaction of Bergmann’s rule with migration and nest location.**

a) Latitude (n=513 species)

| **Migration** | **Nest location** | **Mean *β*** | ***β* 95% CI** | ***pMCMC*** | **Mean *h^2^*** | ***h^2^* 95% CI** |
| --- | --- | --- | --- | --- | --- | --- |
| Sedentary | Open | 0.009 | [0.002, 0.016] | 0.025 | 0.989 | [0.971, 0.998] |
|  | Semi | 0.006 | [0.002, 0.010] | 0.004 |  |  |
|  | Enclosed | 0.003 | [-0.004, 0.011] | 0.354 |  |  |
| Short | Open | -0.002 | [-0.007,0.003] | 0.534 |  |  |
|  | Semi | 0.001 | [-0.005, 0.006] | 0.768 |  |  |
|  | Enclosed | <-0.001 | [-0.010, 0.010] | 0.923 |  |  |
| Long | Open | <-0.001 | [-0.007 0.006] | 0.902 |  |  |
|  | Semi | -0.001 | [-0.007, 0.005] | 0.629 |  |  |
|  | Enclosed | 0.003 | [-0.008, 0.015] | 0.668 |  |  |

b) Temperature (n=515 species)

| **Migration** | **Nest location** | **Mean *β*** | ***β* 95% CI** | ***pMCMC*** | **Mean *h^2^*** | ***h^2^* 95% CI** |
| --- | --- | --- | --- | --- | --- | --- |
| Sedentary | Open | -0.013 | [-0.026, -0.002] | 0.029 | 0.989 | [0.970, 0.999] |
|  | Semi | -0.017 | [-0.026, -0.009] | <0.001 |  |  |
|  | Enclosed | -0.009 | [-0.024, 0.005] | 0.233 |  |  |
| Short | Open | 0.001 | [-0.008, 0.010] | 0.795 |  |  |
|  | Semi | -0.004 | [-0.015, 0.007] | 0.476 |  |  |
|  | Enclosed | -0.002 | [-0.023, 0.017] | 0.838 |  |  |
| Long | Open | -0.003 | [-0.015, 0.010] | 0.681 |  |  |
|  | Semi | <0.001 | [-0.015, 0.015] | 0.924 |  |  |
|  | Enclosed | <0.001 | [-0.032, 0.031] | 0.996 |  |  |

Results of models allowing the slope of body mass on a) latitude or b) temperature to vary between species with different migratory strategies (sedentary, short- or long-distance) and nest locations (open, semi-open or enclosed). Mean *β* / *β* 95% CI / *pMCMC* = mean regression slope estimates, 95% credible intervals and pMCMC values, mean *h^2^ / h^2^* 95% CI = mean heritability (phylogenetic signal) and 95% credible intervals.

**Figure S1.**

Comparison of one of our key results between models with different prior specifications for the fixed effects. The posterior densities are for the effect of latitude on body mass for open-nesting, non-migratory species from the full model including latitude, nest design and migration interactions as predictors. In our original analyses (a.) we used highly diffuse normal prior distributions for the fixed effects, with a mean of zero and variance of 10^8^. Following the suggestion in (1) that large variances in fixed effects priors can have unintended consequences, we re-ran this model with smaller variances of 10^4^ (b.) and 10^2^ (c.). We found that these alternative prior specifications had no material effects on the posterior distributions.

**Figure S2.**

Comparison of one of our key results between models with different prior specifications for the phylogenetic effects. The posterior densities are for phylogenetic random effects from the full model including latitude, nest design and migration interactions as predictors. In our original analyses (a.) we used an inverse-Wishart prior for the phylogenetic variance, with V=1 and nu=0.002, equivalent to an inverse Gamma distribution with shape and scale parameters set to 0.001, resulting in a near-uniform prior. Following the suggestion in (1) that flat priors can have unintended consequences, we re-ran this model more informative priors setting nu to 0.02 (b.) and 0.2 (c.). We found that these alternative prior specifications had no material effects on the posterior distributions.

**Figure S3.**

Species’ body mass against a) breeding latitude midpoint and b) breeding range mean temperature across the whole sample. Mean slopes from the posterior distributions are indicated by thick, opaque lines, while slopes from the entire posterior distribution are plotted as thin, semi-transparent lines.

**Bibliography**

1. Banner KM, Irvine KM, Rodhouse TJ (2020) The use of Bayesian priors in Ecology: The good, the bad and the not great. *Methods in Ecology and Evolution* 11(8):882–889.
